# Supplementary material for: Associations between In-Hospital Mortality and Prescribed Parenteral Energy and Amino Acid Doses in Critically Ill Patients: A Retrospective Cohort Study Using a Medical Claims Database
Source: Nutrients. 2023 Dec 24;16(1):57. doi: 10.3390/nu16010057 (PMC10781052; doi:10.3390/nu16010057)
Supplement: Supplementary file 1 [file nutrients-16-00057-s001.zip › nutrients-2772160-supplementary.pdf]

Supplementary Material Table S1 Classification of primary diagnoses

Diagnosis	International Classification of Diseases, 10th revision (ICD-10) Codes
Sepsis	A02.1, A20.7, A22.7, A26.7, A32.7, A40-A41, A42.7, B37.7
Neoplasms	C00-D48, excluding D10-D36
Diseases of the nervous system	G00-G99
Ischemic heart disease	I20-I25
Heart failure	I50
Cerebrovascular diseases	I60-I69
Other circulatory system diseases	I00-I49, I51-I52, I70-I99
Pneumonia	J10.0、J11.0、J12-J18、J85.1
Interstitial respiratory diseases	J80-J84
Other respiratory diseases	J00-J09、J10.1-J10.8、J11.1-J11.8、J20-J70、J85.0、J85.2-J85.3、J86-J99
Diseases of the digestive system	K00-K93
Kidney diseases	N00-N29
Injury, poisoning and certain other consequences of external causes	S00-S99, T00-T14, T20-T65
Other	Other than above

Supplementary Material Table S2 Classification of surgical procedures

Category of Surgery	Japan-specific Surgical Codes
Cardiovascular	538-K6105, K613-K6173, K619-628, Excluding K601 and K602
Gastroenterological	K5201-K5372, K630-K663, K665-K7424
Cerebrovascular	K145-K181
Respiratory	K488-K5181
Orthopedic	K023-K144
Urological/Gynecological	K757-K8903
Multiple surgeries	More than one surgery in categories above
Other	Excluding above categories, K601, and K602
No surgery	Not applicable to any of codes above, or **no** record of surgery under general or lumber spinal anesthesia

Supplementary Material Table S3 Characteristics of 20,773 adult ICU patients hospitalized from January 2010 through June 2020 in Japan.

Characteristics		Patient Groups Based on Energy Dose[a]			Patient Groups Based on Amino Acid Dose[b]		
		Very low-calorie	Low-calorie	Moderate-calorie	Very low-amino acid	Low-amino acid	Moderate-amino acid
		n=10,384	n=7,103	n=3,286	n=10,908	n=5,836	n=4,029
**Beds in admission hospital**, n (%)	< 200	346 (3.3)	246 (3.5)	93 (2.8)	395 (3.6)	216 (3.7)	74 (1.8)
	≥ 200 to < 500	5,685 (54.7)	4,146 (58.4)	2,045 (62.2)	5,908 (54.2)	3,514 (60.2)	2,454 (60.9)
	≥ 500	4,353 (41.9)	2,711 (38.2)	1,148 (34.9)	4,605 (42.2)	2,106 (36.1)	1,501 (37.3)
**Admission years**, n (%)	2010–2011	285 (2.7)	260 (3.7)	163 (5.0)	320 (2.9)	193 (3.3)	195 (4.8)
	2012–2013	942 (9.1)	702 (9.9)	472 (14.4)	1,006 (9.2)	588 (10.1)	522 (13.0)
	2014–2015	2,081 (20.0)	1,460 (20.6)	768 (23.4)	2,148 (19.7)	1,201 (20.6)	960 (23.8)
	2016–2017	3,042 (29.3)	2,115 (29.8)	882 (26.8)	3,151 (28.9)	1,777 (30.4)	1,111 (27.6)
	2018–2019	3,473 (33.4)	2,242 (31.6)	850 (25.9)	3,683 (33.8)	1,801 (30.9)	1,081 (26.8)
	2020	561 (5.4)	324 (4.6)	151 (4.6)	600 (5.5)	276 (4.7)	160 (4.0)
**Japan Coma Scale**, n (%)	0	4,184 (40.3)	3,865 (54.4)	1,981 (60.3)	4,739 (43.4)	2,938 (50.3)	2,353 (58.4)
	1–3	1,749 (16.8)	1,231 (17.3)	561 (17.1)	1,805 (16.5)	1,069 (18.3)	667 (16.6)
	10–30	1,037 (10.0)	618 (8.7)	243 (7.4)	993 (9.1)	594 (10.2)	311 (7.7)
	100–300	3,413 (32.9)	1,389 (19.6)	501 (15.2)	3,370 (30.9)	1,235 (21.2)	698 (17.3)
	NA	1 (0.0)	0 (0.0)	0 (0.0)	1 (0.0)	0 (0.0)	0 (0.0)
	Cardiovascular	992 (9.6)	690 (9.7)	296 (9.0)	1,280 (11.7)	464 (8.0)	234 (5.8)

Surgery[c] n (%)	Gastroenterological	1,356 (13.1)	2,034 (28.6)	1,102 (33.5)	1,519 (13.9)	1,412 (24.2)	1,561 (38.7)
	Cerebrovascular	840 (8.1)	355 (5.0)	165 (5.0)	738 (6.8)	356 (6.1)	266 (6.6)
	Respiratory	25 (0.2)	24 (0.3)	21 (0.6)	26 (0.2)	16 (0.3)	28 (0.7)
	Orthopedic	25 (0.2)	34 (0.5)	13 (0.4)	34 (0.3)	23 (0.4)	15 (0.4)
	Urological/Gynecological	52 (0.5)	34 (0.5)	18 (0.5)	45 (0.4)	35 (0.6)	24 (0.6)
	Multiple surgeries	30 (0.3)	37 (0.5)	36 (1.1)	36 (0.3)	25 (0.4)	42 (1.0)
	Other	118 (1.1)	164 (2.3)	84 (2.6)	144 (1.3)	104 (1.8)	118 (2.9)
	No surgery	6,946 (66.9)	3,731 (52.5)	1,551 (47.2)	7,086 (65.0)	3,401 (58.3)	1,741 (43.2)

[a] Groups based on mean daily parenteral energy dose days 4 through 7: Very low-calorie (< 10 kcal/kg/day), Low-calorie (≥ 10 and < 20 kcal/kg/day), Moderate-calorie (≥ 20 kcal/kg/day).

[b] Groups based on mean daily amino acid dose days 4 through 7: Very low-amino acid (< 0.3 g/kg/day), Low-amino acid (≥ 0.3 and < 0.6 g/kg/day), Moderate-amino acid (≥ 0.6 g/kg/day).

[c] Surgeries performed under general or lumbar spinal anesthesia between the day of hospital admission and the day of ICU admission.

Abbreviations: ICU, intensive care unit; NA, not available (i.e., data for calculation unavailable).

Supplementary Material Figure S1 Change in median dose of amino acids, lipid, and carbohydrate in each group[a] (A) and change in median dose of energy, lipid, and carbohydrate in each group[b] (B), prescribed in parenteral nutrition during days 1 through 7 for 20,773 adult ICU patients hospitalized from January 2010 through June 2020 in Japan.

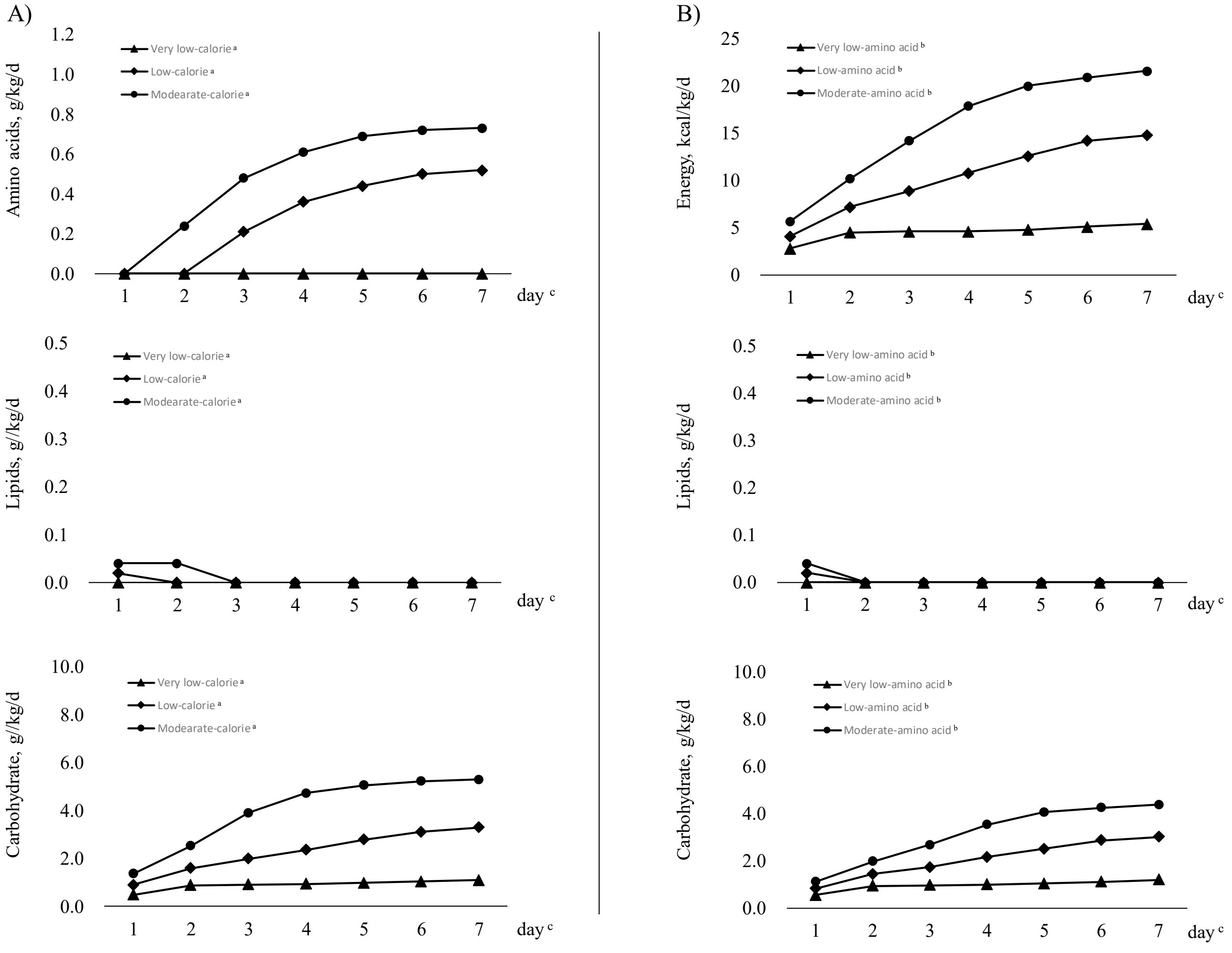

[a] Groups based on mean daily parenteral energy dose days 4 through 7: Very low-calorie (< 10 kcal/kg/day); n = 10,384, Low-calorie (≥ 10 and < 20 kcal/kg/day): n = 7,103, and Moderate-calorie (≥ 20 kcal/kg/day): n = 3,286.
[b] Groups based on mean daily parenteral amino acid dose days 4 through 7: Very low-amino acid (< 0.3 g/kg/day); n =10,908, Low-amino acid (≥ 0.3 and < 0.6 g/kg/day); n = 5,836, and Moderate-amino acid (≥ 0.6 g/kg/day); n = 4,029.
[c] Day 1 regarded as the day of ICU admission.
